# Prenatal diagnosis of chromosomal aberrations by chromosomal microarray analysis in foetuses with ventriculomegaly

**DOI:** 10.1038/s41598-020-77400-8

**Published:** 2020-11-27

**Authors:** Jiamin Wang, Zhu Zhang, Qinqin Li, Hongmei Zhu, Yi Lai, Wei Luo, Shanling Liu, He Wang, Ting Hu

**Affiliations:** 1 Department of Obstetrics and Gynaecology, West China Second University Hospital, Sichuan University, No. 20, Section 3, Renminnan Road, Chengdu, 610041 Sichuan China; 2 Key Laboratory of Birth Defects and Related Diseases of Women and Children (Sichuan University), Ministry of Education, Chengdu, China

**Keywords:** Chromosomes, Clinical genetics

## Abstract

Ventriculomegaly is considered to be linked to abnormal neurodevelopment outcome. The aim of this retrospective study was to investigate the current applications of chromosomal microarray analysis (CMA) in foetuses with ventriculomegaly. A total of 548 foetuses with ventriculomegaly detected by prenatal ultrasound underwent single nucleotide polymorphism (SNP) array testing and were subjected to long-term follow-up. The overall prevalence of chromosomal aberrations was 7.30% (40/548), including 4.20% (23/548) with pathogenic/likely pathogenic copy number variants. The incidence of chromosomal aberrations was significantly higher in foetuses with bilateral ventriculomegaly than in those with unilateral ventriculomegaly (10.56% vs. 5.71%, *P* = 0.040), in foetuses with non-isolated ventriculomegaly than in those with isolated ventriculomegaly (12.99% vs. 2.38%, *P* < 0.0001), and in foetuses with severe ventriculomegaly than in those with mild-to-moderate ventriculomegaly (23.08% vs. 6.51%, *P* = 0.005). The outcome in foetuses with mild ventriculomegaly was significantly better than in those with moderate ventriculomegaly (95.60% vs. 84.00%, *P* = 0.003). Thus, CMA should be regarded as the first-tier test for prenatal diagnosis of foetal ventriculomegaly, especially in foetuses with bilateral or non-isolated ventriculomegaly. The outcome of foetuses with mild ventriculomegaly is favourable; however, there is an increased risk of neurodevelopmental disabilities in foetuses with moderate ventriculomegaly.

## Introduction

Foetal ventriculomegaly, defined as dilation of the cerebral ventricles (atrial diameter ≥ 10 mm), is a common cerebral anomaly on prenatal ultrasound, with an estimated prevalence of 0.3–1.5 per 1000 births^1^. Foetal ventriculomegaly is typically categorized as mild (10–12 mm), moderate (13–15 mm), or severe (> 15 mm) ventriculomegaly^2^. Foetal ventriculomegaly can also be classified as unilateral or bilateral ventriculomegaly as well as isolated or non-isolated ventriculomegaly^3^.

Foetuses with severe ventriculomegaly are known to have a poor prognosis in accordance with survival and neurodevelopmental outcome^4^. However, the prognosis for infants with mild-to-moderate ventriculomegaly is widely variable, which makes genetic counselling quite challenging in clinical practice. The prognosis depends on whether it is combined with structural abnormalities, especially central nervous system (CNS) anomalies, chromosomal aberrations, congenital infections of cytomegalovirus (CMV) or toxoplasmosis (TOX), and the progression of the ventricular dilation^5^.

Therefore, in this retrospective study, which comprehensively focused on aetiology, we systematically investigated the chromosomal aberrations of foetal ventriculomegaly by single nucleotide polymorphism (SNP) array, the additional CNS anomalies by magnetic resonance imaging (MRI), and the status of congenital foetal CMV infections. Furthermore, pregnancy outcomes were evaluated based on the follow-up information on foetuses with different types of ventriculomegaly.

## Results

We analysed a total of 548 foetuses with ventriculomegaly by SNP array. The maternal age was between 18 and 46 years (median = 28.68 ± 4.49 years); 10.58% (58/548) of the gravidas were of advanced maternal age (35 years or older at delivery); 60.95% (334/548) of the gravidas were nulliparous; and the gestational age (GA) ranged from 22 to 34^+5^ weeks (median = 27.57 ± 5.78 weeks). The demographic and clinical characteristics of the gravidas is shown in Supplementary Table S1.

There were 509 (92.88%) gravidas who underwent foetal MRI of the CNS after 26 weeks GA. Eighteen (3.54%, 18/509) cases with additional CNS anomalies were detected, including 6 with agenesis of the corpus callosum, 5 with arachnoid cysts, 3 with hypoplasia of the corpus callosum, 2 with Dandy–Walker malformation, 1 with migrational abnormality, and another with holoprosencephaly.

In the 3 gravidas with serous positive IgM and low IgG avidity of CMV, CMV infection was confirmed in 2 cases by polymerase chain reaction (PCR) testing of amniotic fluid. As regards the 2 foetuses, one had moderate bilateral ventriculomegaly, hyperechogenic bowel, and hepatic calcifications, whereas the other had moderate bilateral ventriculomegaly and pericardial effusion.

### Prevalence of chromosomal aberrations in foetal ventriculomegaly

The overall prevalence of chromosomal aberrations in foetuses with ventriculomegaly was 7.30% (40/548) (Table 1). Among all cases, numerical chromosomal aberrations were identified in 17 (3.10%), including 1 triploid and 3 with mosaic trisomy (Table 2). In addition, 23 (4.20%) foetuses were identified with pathogenic/likely pathogenic (P/LP) copy number variants (CNVs) and 2 (0.36%) with uncertain clinical significance (VUS).

**Table 1:** Summary of 750 K SNP array results in 548 fetuses with ventriculomegaly.

Characteristics	SNP array results (%)				Total
	Aneuploidies/polyploidy	P/LP CNVs	VUS	Normal	
**Lateral of ventriculomegaly**					
Unilateral	9 (2.45)	12 (3.26)	1 (0.27)	346 (94.02)	368
Bilateral	8 (4.44)	11 (6.11)	1 (0.56)	160 (88.89)	180
**Ultrasound findings**					
Isolated ventriculomegaly	4 (1.36)	3 (1.02)	1 (0.34)	286 (97.28)	294
Non-isolated ventriculomegaly	13 (5.12)	20 (7.87)	1 (0.39)	220 (86.62)	254
**Degrees of ventriculomegaly**					
Mild	9 (2.05)	17 (3.87)	2 (0.46)	411 (93.62)	439
Moderate	5 (6.02)	3 (3.61)	0 (0.00)	75 (90.36)	83
Severe	3 (11.54)	3 (11.54)	0 (0.00)	20 (76.92)	26

*P/LP CNVs* pathogenic/likely pathogenic copy number variants, *VUS* variant of uncertain significance.

**Table 2:** Characteristics of fetuses with aneuploidies/polyploidy detected by 750 K SNP array among the 548 fetuses with ventriculomegaly.

No	SNP array results	Degrees of ventriculomegaly	Other ultrasound findings	Outcome
1	Trisomy 21	Mild (bilateral)	–	TOP
2	Trisomy 21	Moderate (bilateral)	–	TOP
3	Trisomy 21	Mild (unilateral)	Hypoplastic nasal bone	TOP
4	Trisomy 21	Mild (unilateral)	Hypoplastic nasal bone	TOP
5	Trisomy 21	Mild (bilateral)	Enlarged cisterna magna, intracardiac echogenic focus	TOP
6	Trisomy 21	Moderate (bilateral)	Intracardiac echogenic focus	TOP
7	Trisomy 21	Moderate (bilateral)	short femur length	TOP
8	Trisomy 21	Mild (unilateral)	Pleural effusion, fetal hydrops	TOP
9	Trisomy 21	Mild (unilateral)	Tetralogy of Fallot, pericardial effusion	TOP
10	Trisomy 21	Mild (unilateral)	Echogenic kidney	TOP
11	Trisomy 18	Servere (bilateral)	–	Intrauterine fetal death
12	Trisomy 18	Servere (bilateral)	VSD, talipes equinovarus, polyhydramnios	TOP
13	XXY	Moderate (unilateral)	short femur length	TOP
14	Triploid	Servere (unilateral)	IUGR, cervical lymphadenoma	TOP
15	Mosaic trisomy 9	Mild (unilateral)	Cerebellar vermis hypoplasia	TOP
16	Mosaic trisomy 9	Mild (bilateral)	C4–C5 vertebral abnormality, pyelectasis	TOP
17	Mosaic trisomy 12	Mild (unilateral)	–	TOP

*VSD* ventricular septal defect, *IUGR* intrauterine growth retardation, *TOP* termination of pregnancy.

In the 23 foetuses with P/LP CNVs, 14 were identified with deletions/duplications larger than 5 Mb, and these were also detected with karyotyping (Table 3). Karyotyping detected subtelomeric aberrations in 5 cases, including 3 confirmed cases (foetuses 30, 31, and 32) and 2 missed cases (foetuses 33 and 37). Furthermore, the 3 confirmed cases (foetuses 30, 31, and 32) were confirmed to be inherited from normal parents with balanced translocations. Among the other 9 cases subjected to karyotyping, 1 was missed (foetus 35) and 3 (foetuses 21, 26, and 40) with GA exceeding 32 weeks had cell culture failure.

**Table 3:** Characteristics of fetuses with P/LP CNVs detected by 750 K SNP array among the 548 fetuses with ventriculomegaly.

No	Degrees of ventriculomegaly	Other ultrasound findings	P/LP CNVs (GRCh37)	Size of CNVs (kb)	Copy number	Known syndromes	OMIM gene	Inherited or de novo	karyotyping	Outcome
18	Mild (unilateral)	–	arr 3q29 (195793334_197520553)x3	1727	Gain	3q29 microduplication syndrome	–	de novo	–	TOP
19	Mild (bilateral)	–	arr 7q11.23 (72589455_74184702)x3	1595	Gain	7q11.23 duplication syndrome	–	de novo	–	TOP
20	Mild (bilateral)	–	arr Xp22.31 (6458940_8135568)x0	1677	Loss	steroid sulphatase deficiency (STS)	STS	Inherited from normal mother	–	TOP
21	Mild (unilateral)	Intracardiac echogenic focus	arr 1p12q32.2 (118269042_210703926)x3	66,772	Gain	1q21.1 recurrent microduplication (possible susceptibility locus for neurodevelopmental disorders)	–	de novo	Failure of karyotyping	TOP
22	Mild (bilateral)	Short femur length	arr 16p11.2 (29581101_30190029)x3	609	Gain	16p11.2 microduplication syndrome	–	de novo	–	TOP
23	Mild (bilateral)	Polyhydramnios	arr 16p13.12p12.3 (14512852_17637607)x3	3125	Gain	16p13.11 recurrent microduplication (neurocognitive disorder susceptibility locus)	–	Inherited from normal mother	–	Born
24	Mild (unilateral)	Echogenic kidney	arr 17q12 (34822465_36307773)x1	1485	Loss	17q12 recurrent deletion syndrome/ RCAD (renal cysts and diabetes)	HNF1B	de novo	–	TOP
25	Mild (unilateral)	Cerebral dysplasia	arr 22q11.21 (18919477_21454872)x3	2535	Gain	22q11 duplication syndrome	–	de novo	–	TOP
26	Mild (unilateral)	Short femur, hydronephrosis, abnormality of the hands, single umbilical artery, polyhydramnios	arr 1p31.3p22.1 (68767187_92069100)x1	23,302	Loss	–	–	de novo	Failure of karyotyping	TOP
27	Mild (bilateral)	Short femur length, absent gallbladder, polyhydramnios	arr 9p24.3q13 (208454_68188534)x3	38,579	Gain	–	–	de novo	46,XX,add(21)(p11.2)	TOP
28	Mild (bilateral)	Hypertelorism	arr 13q22.2q34 (76253450_115107733)x3	38,854	Gain	–	–	de novo	46,XY,add(13)(p11.2)	TOP
29	Mild (unilateral)	Echogenic bowel, intracardiac echogenic focus	arr 18q22.1q23 (63244135_78013728)x1	14,770	Loss	–	–	de novo	46,XX,del(18)(q22)	TOP
30	Mild (unilateral)	VSD, hydronephrosis	arr 1p36.33p36.32 (849466_2579267)x1	1730	Loss	–	GNB1	Balanced translocation of normal mother	46,XY,der(1)t(1;15)(p36;q25)(mat)	TOP
			arr 15q25.1q26.3 (80800155_102429040)x3	21,629	Gain	15q26 overgrowth syndrome	–			
31	Mild (bilateral)	Ascites, pyelectasis, echogenic bowel, single umbilical artery	arr 2q32.2q37.3 (190153126_242782258)x3x1	52,629	Gain	–	–	Balanced translocation of normal father	46,XX,der(4)t(2;4)(q32;q34)(pat)	TOP
			arr 4q34.3q35.2 (177791395_190957460)	13,166	Loss	–	–			
32	Mild (unilateral)	Enlarged cisterna magna, Tetralogy of Fallot, PLSVC	arr 8p23.3p22 (158048_17861146)x3	17,703	Gain	8p23.1 duplication syndrome	–	Balanced translocation of normal father	47,XY,der(9)t(8;9)(p22;q22)(pat)	TOP
			arr 9p24.3q22.2 (208454_91880960)x3	91,673	Gain	–	–			
33	Mild (unilateral)	Absent gallbladder, intracardiac echogenic focus	arr 12p13.33 (173786_1606351)x1	1433	Loss	12p13.33 microdeletion syndrome	–	de novo	46,XX (missed by karyotyping)	TOP
			arr 20q13.2q13.33 (52342290_62913645)x3	10,571	Gain	–	–			
34	Mild (unilateral)	Short femur length, mitral valve regurgitation	arr Xp22.33p11.1 (2564409_58227320)x1	55,663	Loss	–	–	de novo	45,X [16]/ 46,X,del(X)(p10) [14]	TOP
			arr Xq11.1q28 (62036670_154973155)x1.5	92,936	Loss (Mosaic)	–	–			
35	Moderate (unilateral)	Hypoplasia of the corpus callosum	arr 6q25.3q27 (160569492_170914297)x1	10,345	Loss	–	ERMARD	de novo	46,XX (missed by karyotyping)	TOP
36	Moderate (unilateral)	Talipes equinus	arr 12q24.21 (116025185_116416736)x1	392	Loss	–	MED13L	de novo	–	TOP
37	Moderate (bilateral)	Hypoplasia of the corpus callosum	arr 3q28q29 (190376787_197851444)x3	7475	Gain	3q29 microduplication syndrome	PAK2	de novo	46,XY (missed by karyotyping)	TOP
			arr 17p13.3 (525_2780094)x1	2780	Loss	Miller–Dieker syndrome (MDS)	PAFAH1B1			
38	Severe (bilateral)	Hypoplasia of the corpus callosum	arr 22q11.21 (18919477_21800471)x3	2881	Gain	22q11 duplication syndrome	–	de novo	–	TOP
39	Severe (bilateral)	Hydronephrosis	arr 5p15.33p15.31 (113576_9308549)x3	9195	Gain	Cri-du-chat Syndrome (5p deletion)	–	de novo	46,XX,del(5)(p15)	TOP
40	Severe (bilateral)	Cerebellar hypoplasia, enlarged cisterna magna, Cystic hygroma, cleft lip	arr 12p12.3q12 (19580413_44341874)x3	24,761	Gain	–	PTHLH	de novo	Failure of karyotyping	TOP
			arr 13q33.2q34 (106367669_115107733)x4	8740	Gain	–	–			

*P/LP CNVs* pathogenic/likely pathogenic copy number variants, *VSD* ventricular septal defect, *PLSVC*: persistent left superior vena cava; *TOP* termination of pregnancy.

Thirteen types of microdeletion/microduplication syndromes were identified in 14 cases, including 1q21.1 recurrent microduplication, 3q29 microduplication syndrome, Cri-du-chat syndrome, 7q11.23 duplication syndrome, 8p23.1 duplication syndrome, 12p13.33 microdeletion syndrome, 15q26 overgrowth syndrome, 16p13.11 recurrent microduplication, 16p11.2 microduplication syndrome, Miller–Dieker syndrome (MDS), 17q12 recurrent deletion syndrome, 22q11 duplication syndrome, and steroid sulphatase deficiency (STS). Additionally, 3 cases were further confirmed by karyotyping among 5 cases with deletions/duplications > 5 Mb (Table 3).

### Incidence of chromosomal aberrations in unilateral vs bilateral ventriculomegaly

**Figure 1:**
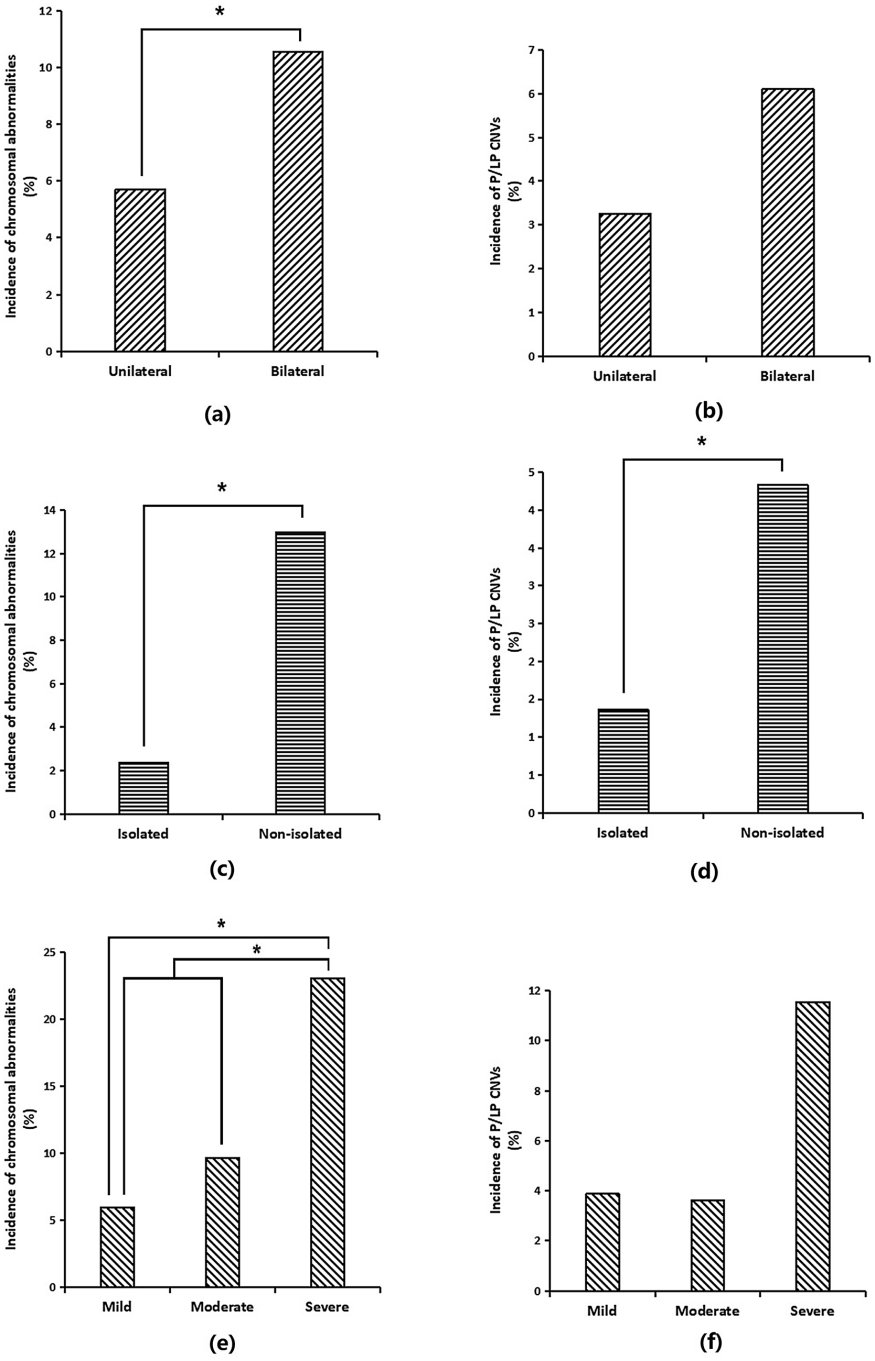
The incidences of chromosomal aberrations in subgroups of foetuses with ventriculomegaly. (**a**) The incidence of chromosomal aberrations in the unilateral ventriculomegaly group [5.71% (21/368)] was significantly lower than in the bilateral ventriculomegaly group [10.56% (19/180)] (*P* = 0.040); (**b**) the incidences of P/LP CNVs were not significantly different between the unilateral ventriculomegaly group [3.26% (12/368)] and bilateral ventriculomegaly group [6.11% (11/180)] (*P* = 0.118); (**c**) the incidence of chromosomal aberrations in the isolated ventriculomegaly group [2.38% (7/294)] was significantly lower than in the non-isolated ventriculomegaly group [12.99% (33/254)] (*P* < 0.0001); (**d**) the incidence of P/LP CNVs in the isolated ventriculomegaly group [1.36% (4/294)] was significantly lower than in the non-isolated ventriculomegaly group [7.48% (19/254)] (*P* < 0.0001); (**e**) The incidence of chromosomal aberrations in the mild ventriculomegaly group [5.92% (26/439)] was significantly lower than in the severe ventriculomegaly group [23.08% (6/26)] (*P* = 0.003), whereas the incidence in the mild-to-moderate ventriculomegaly group [6.51% (34/522)] was significantly lower than that in the severe ventriculomegaly group [23.08% (6/26)] (*P* = 0.005); (**f**) The incidences of P/LP CNVs were not significantly different among the mild ventriculomegaly [3.87% (17/439)], moderate ventriculomegaly [3.61% (3/83)], and severe ventriculomegaly [11.54% (3/26)] groups (mild vs. moderate ventriculomegaly, *P* = 1.000; mild ventriculomegaly vs. severe ventriculomegaly, *P* = 0.169; and moderate ventriculomegaly vs. severe ventriculomegaly, *P* = 0.146).

The incidences of chromosomal aberrations in foetuses with unilateral ventriculomegaly and bilateral ventriculomegaly were 5.71% (21/368) and 10.56% (19/180), respectively (Table 1). The incidence in foetuses with unilateral ventriculomegaly was significantly lower than in those with bilateral ventriculomegaly (*P* = 0.040) (Fig. 1a). However, the incidences of P/LP CNVs were not significantly different between these groups [3.26% (12/368) vs. 6.11% (11/180), *P* = 0.118] (Fig. 1b).

### Incidence of chromosomal aberrations in isolated vs non-isolated ventriculomegaly

The incidences of chromosomal aberrations were 2.38% (7/294) and 12.99% (33/254) in foetuses with isolated and non-isolated ventriculomegaly, respectively (Table 1). The incidence in foetuses with isolated ventriculomegaly was significantly lower than in those with non-isolated ventriculomegaly (*P* < 0.0001) (Fig. 1c). Likewise, the incidence of P/LP CNVs in foetuses with isolated ventriculomegaly was significantly lower than in those with non-isolated ventriculomegaly [1.36% (4/294) vs.7.48% (19/254), *P* < 0.0001] (Fig. 1d).

### Subgroup analysis of different severities of foetal ventriculomegaly

The incidences of chromosomal aberrations were 5.92% (26/439), 9.64% (8/83), and 23.08% (6/26) in foetuses with mild, moderate, and severe ventriculomegaly, respectively (Table 1). The incidence in foetuses with mild ventriculomegaly was significantly lower than in those with severe ventriculomegaly (5.92% vs. 23.08%, *P* = 0.003), whereas that in foetuses with mild-to-moderate ventriculomegaly was significantly lower than in those with severe ventriculomegaly [6.51% (34/522) vs. 23.08% (6/26 ), *P* = 0.005]. However, statistical analysis showed that these incidences were not significantly different between foetuses with mild ventriculomegaly and those with moderate ventriculomegaly (5.92% vs. 9.64%, *P* = 0.208) or between foetuses with moderate ventriculomegaly and those with severe ventriculomegaly (9.64% vs. 23.08%, *P* = 0.147) (Fig. 1e).

The incidences of P/LP CNVs were 3.87% (17/439), 3.61% (3/83), and 11.54% (3/26) in foetuses with mild, moderate, and severe ventriculomegaly, respectively. However, statistical analysis showed that these incidences were not significantly different from each other (mild vs. moderate ventriculomegaly, *P* = 1.000; mild vs. severe ventriculomegaly, *P* = 0.169; and moderate vs. severe ventriculomegaly, *P* = 0.146) (Fig. 1f).

### The clinical follow-up assessments

Clinical follow-up assessments were performed on 495 (90.33%) cases (Table 4). Except for the foetus (foetus 23) confirmed with 16p13.11 recurrent microduplication (neurocognitive disorder susceptibility locus) inherited from normal mother and whose mild bilateral ventriculomegaly was resolved at 33 GA resulting in a favourable outcome at birth, the other 39 foetuses with chromosomal aberrations all underwent termination of pregnancy (TOP). The ventriculomegaly of one foetus with VUS progressing from mild to severe underwent TOP at 34 GA.

**Table 4:** Clinical follow-up assessment of the 548 fetuses with ventriculomegaly.

Ultrasound category	SNP array results												Total
	Aneuploidies/polyploidy or P/LP CNVs (n(%))				VUS (n(%))				Normal (n(%))				
	Normal infants	TOP/ Intrauterine fetal death	Birth with defects	Loss of follow-up	Normal infants	TOP/ Intrauterine fetal death	Birth with defects	Loss of follow-up	Normal infants	TOP/ Intrauterine fetal death	Birth with defects	Loss of follow-up	
**Isolated ventriculomegaly**	**–**	**7 (100.00)**	**–**	**–**	**–**	**1 (100.00)**	**–**	**–**	**226 (79.02)**	**22 (7.69)**	**10 (3.50)**	**28 (9.79)**	**294**
Mild	–	5 (100.00)	–	–	–	1 (100.00)	–	–	198 (84.99)	6 (2.57)	6 (2.57)	23 (9.87)	239
Moderate	–	1 (100.00)	–	–	–	–	–	–	28 (60.87)	9 (19.57)	4 (8.69)	5 (10.87)	47
Severe	–	1 (100.00)	–	–	–	–	–	–	–	7 (100.00)	–	–	8
**Non-isolated ventriculomegaly**	**1 (3.03)**	**32 (96.97)**	**–**	**–**	**1 (100.00)**	**–**	**–**	**–**	**142 (64.55)**	**40 (18.18)**	**13 (5.91)**	**25 (11.36)**	**254**
Mild	1 (4.76)	20 (95.24)	–	–	1 (100.00)	–	–	–	128 (71.91)	19 (10.67)	9 (5.06)	22 (12.36)	200
Moderate	–	7 (100.00)	–	–	–	–	–	–	14 (48.28)	8 (27.59)	4 (13.79)	3 (10.34)	36
Severe	–	5 (100.00)	–	–	–	–	–	–	–	13 (100.00)	–	–	18
**Total**	**1**^a^	**39**	**–**	**–**	**1**	**1**	**–**	**–**	–	–	–	–	**548**

*P/LP CNVs* pathogenic/likely pathogenic copy number variants, *TOP* termination of pregnancy.

^a^16p13.11 recurrent microduplication (neurocognitive disorder susceptibility locus) (inherited from normal mother).

The rate of normal infant was 81.24% (368/453) in foetuses without chromosomal aberrations, except those lost to follow-up. The 2 cases confirmed with CMV infection underwent TOP. The rates of normal infant in isolated and non-isolated ventriculomegaly group were 95.76% (226/236) and 91.61% (142/155), respectively. The outcomes of foetuses were not significantly different between these groups (*P* = 0.088). In foetuses with isolated ventriculomegaly, the most common reason for TOP was progressive ventriculomegaly. Additional CNS anomalies detected by MRI included agenesis/hypoplasia of the corpus callosum, Dandy–Walker malformation, migrational abnormality, and holoprosencephaly. Meanwhile, as all the 20 foetuses with severe ventriculomegaly underwent TOP, the rates of normal infant in the mild and moderate ventriculomegaly groups were 95.60% (326/341) and 84.00% (42/50), respectively. The outcome of foetuses with mild ventriculomegaly was significantly better than that of those with moderate ventriculomegaly (*P* = 0.003).

Additionally, for foetuses without chromosomal aberrations, except those that underwent TOP or intrauterine foetal death, the rate of foetuses born with clinical defect was 5.88% (23/391). The clinical manifestations included developmental delay, intellectual disability, delayed speech and language development, autism, epilepsy, hyperactivity disorder, hydrocephalus, etc. All these infants were diagnosed with mild-to-moderate ventriculomegaly that never aggravated to severe ventriculomegaly. The rate of clinical defects after birth in the mild ventriculomegaly group was significantly lower than in the moderate ventriculomegaly group [4.40% (15/341) vs. 16.00% (8/50), *P* = 0.003]; however, it was not significantly different between the isolated and non-isolated groups [4.24% (10/236) vs. 8.39% (13/155), *P* = 0.088].

## Discussion

The Society for Maternal-Foetal Medicine (SMFM) has recommended that once ventriculomegaly is detected, a thorough evaluation should be performed, including detailed sonographic evaluation of foetal anatomy, amniocentesis for the assessment of chromosomal aberrations, and a workup for foetal infection^5^. However, few studies have comprehensively explored all the causes of ventriculomegaly. In this study, we systemically investigated the aetiological factors, including chromosomal aberrations by SNP array, foetal CNS structural abnormalities by MRI, and foetal CMV infections by PCR in order to improve prenatal diagnosis and clinical counselling.

The prevalence of MRI-detected additional CNS anomalies was 3.54% (18/509), which was lower than the results from previous studies that ranged from 5 to 10%^1, 6, 7^. While more than 80% of cases included in our study had mild ventriculomegaly (10–12 mm), selection bias may explain the difference. In our study, the most common abnormality missed by ultrasound but detected by MRI was agenesis/hypoplasia of the corpus callosum (9/18), which may induce poor outcome. Moreover, another foetus was identified with migrational abnormality, which has rarely been detected by ultrasound. On account of the MRI-detected additional CNS anomalies associated with adverse outcome, pregnancies were terminated, except for the 5 cases with arachnoid cysts. Thus, foetal MRI for CNS has been proven as a useful tool to identify additional structural anomalies, a quality that may lead to modification of pregnancy management.

The overall prevalence of chromosomal aberrations was 7.30% (40/548), comprising 42.50% chromosomal numerical aberrations and 57.50% P/LP CNVs. Further, karyotyping was performed on 14 cases with P/LP CNVs exceeding 5 Mb, including 5 with microdeletion/microduplication syndromes. However, 3 cases, including 2 with subtelomeric aberrations, were missed (foetuses 33, 35, and 37) and 3 at third-trimester had cell culture failure. Combined with those P/LP CNVs < 5 Mb, we estimated that 15 (2.73%) cases with chromosomal aberrations would be misdiagnosed if karyotyping alone was used in our study. Thus, CMA should be preferred to karyotyping as a first-tier test for prenatal diagnosis of foetal ventriculomegaly.

Until recently, several studies have focused on the relationship between foetal ventriculomegaly and chromosomal aberrations and have reported prevalence of chromosomal aberrations ranging from 5.1 to 17.4%^8–14^. We speculate that such a wide range could be due to different distribution of the ventriculomegaly degrees or combined ultrasound abnormalities among different studies. Further subgroup analysis was performed in our study.

Consistent with data presented by Chang et al.^8^, the incidences of chromosomal aberrations in foetuses with bilateral ventriculomegaly (10.56%) was significantly higher than in those with unilateral ventriculomegaly (5.71%). The incidences of both chromosomal aberrations and P/LP CNVs in foetuses with non-isolated ventriculomegaly (12.99% and 7.48%, respectively) were significantly higher than in those with isolated ventriculomegaly (2.38% and 1.02%, respectively). Wang et al.^15^ conducted a review and revealed that the incidence of chromosomal aberrations in foetuses with ventriculomegaly associated with other structural anomalies (9.5–36%) was higher than in those with isolated ventriculomegaly (1.5–12%)^16–18^, with the similar results for the incidence of P/LP CNVs (6.6–37.9% vs. 4–9.5%)^9, 10, 19, 20^. Thus, CMA should be highly recommended for prenatal diagnosis of foetal ventriculomegaly, especially in foetuses with bilateral or non-isolated ventriculomegaly.

Notably, the incidences of chromosomal aberrations in different degrees of foetal ventriculomegaly were still discordant. Several studies reported that the incidence was higher in mild-to-moderate ventriculomegaly than in severe ventriculomegaly^10, 19^; however, contradicting results were reported by others^20, 21^. In our study, the incidence of both chromosomal aberrations and P/LP CNVs in foetuses with severe ventriculomegaly (23.08% and 11.54%, respectively) was higher than in those with mild-to-moderate ventriculomegaly (5.92% and 3.47%, respectively); however, no significant difference was in the P/LP CNV incidences between these two groups. As the number of cases with severe ventriculomegaly was small in our study, large-scale studies are still required to determine the relationship between the incidence of chromosomal aberrations and the degrees of foetal ventriculomegaly.

Several CNVs have been reported to be associated with foetal ventriculomegaly, for example 22q11.2 microdeletion, 15q11.2 microdeletion, 16p13.11 microdeletion, 16p11.2 microdeletion, and 1q21.1 microdeletion^12, 13, 22, 23^. In our study, 13 types of microdeletion/microduplication syndromes were identified in 14 cases. Ten have been recognized as significant contributors to poor neurodevelopmental outcome, including 1q21.1 recurrent microduplication, 3q29 microduplication syndrome, Cri-du-chat syndrome, 7q11.23 duplication syndrome, 8p23.1 duplication syndrome, 12p13.33 microdeletion syndrome, 16p13.11 recurrent microduplication, 16p11.2 microduplication syndrome, Miller–Dieker syndrome (MDS), and 22q11 duplication syndrome. However, whereas 16p13.11 recurrent microduplication has low penetrance^24^, feotus 23 that inherited this CNV form normal mother was born with favourable outcomes. Additionally, a de novo 12q24.2 microdeletion of 392 kb was detected in foetus 36 with unilateral moderate ventriculomegaly and talipes equinovarus, encompassing exons 24–31 of mediator of RNA polymerase II transcription subunit 13-like (*MED13L*) gene (OMIM: 608771). As a haploinsufficient gene, *MED13L* is linked to mental retardation and distinctive facial features, with or without cardiac defects (MIM: 616789). However, it was not sufficiently powered to address the association of specific CNVs with foetal ventriculomegaly.

Postnatal follow-up from 6 months to 3 years after birth was performed in 495 (90.33%) cases in our study. TOP was opted for all the cases with severe ventriculomegaly due to poor prognosis both in terms of survival and neurodevelopmental disorders^3, 4^. The rate of abnormal clinical outcome in foetuses with mild-to-moderate ventriculomegaly was 5.88%, which concurred with previous studies that had rates ranging from 4 to 11%^1, 7, 16, 25, 26^. The main clinical manifestation was neurodevelopmental delay. Furthermore, we compared the clinical outcome in foetuses with mild ventriculomegaly to that in those with moderate ventriculomegaly. Consistent with previous studies^7, 16, 18, 27^, the rate of normal outcome in mild ventriculomegaly (95.60%) was significantly higher than in moderate ventriculomegaly (84.00%). Our results corroborated a previous submission^5^ that the outcome of foetuses with moderate ventriculomegaly is favourable; however, an increased risk of neurodevelopmental disabilities does exist.

This study still has a few limitations. First, we only detected CMV in the amniotic fluid of gravidas with low IgG avidity and positive IgM for CMV; therefore, other cases with CMV infection might have been missed in our study. Congenital TOX infection has also been confirmed to be associated with foetal ventriculomegaly^28, 29^. According to SMFM recommendations^5^, the tests for CMV and TOX should be performed in further studies, regardless of the history of exposure or the presence of symptoms. Second, a number of single-gene disorders have been associated with ventriculomegaly, such as L1 cell adhesion molecule (*L1CAM*)-associated hydrocephalus, Pettigrew syndrome, Walker–Warburg syndrome, Meckel syndrome, Joubert syndrome, etc.^30, 31^. These conditions have always been associated with severe ventriculomegaly and mostly untreatable; thus, genetic diagnoses are crucial for the parents to assess the risk of recurrence and to facilitate prenatal or preimplantation diagnosis when planning the next pregnancy. Gene sequencing, as a useful approach to remedy the shortage under this condition, should be implemented in future studies.

## Conclusion

When a foetal ventriculomegaly is confirmed by ultrasound, CMA should be regarded as the first-tier test for prenatal diagnosis, especially in foetuses with bilateral ventriculomegaly and non-isolated ventriculomegaly. Tests for infection such as CMV with amniotic fluid samples should be considered in combination with prenatal CMA. MRI is a vital tool in identifying additional CNS anomalies that are associated with adverse outcome. The outcome of foetuses with mild ventriculomegaly is favourable; nonetheless, there is an increased risk of neurodevelopmental disabilities in foetuses with moderate ventriculomegaly.

## Methods

### Subjects

**Figure 2:**
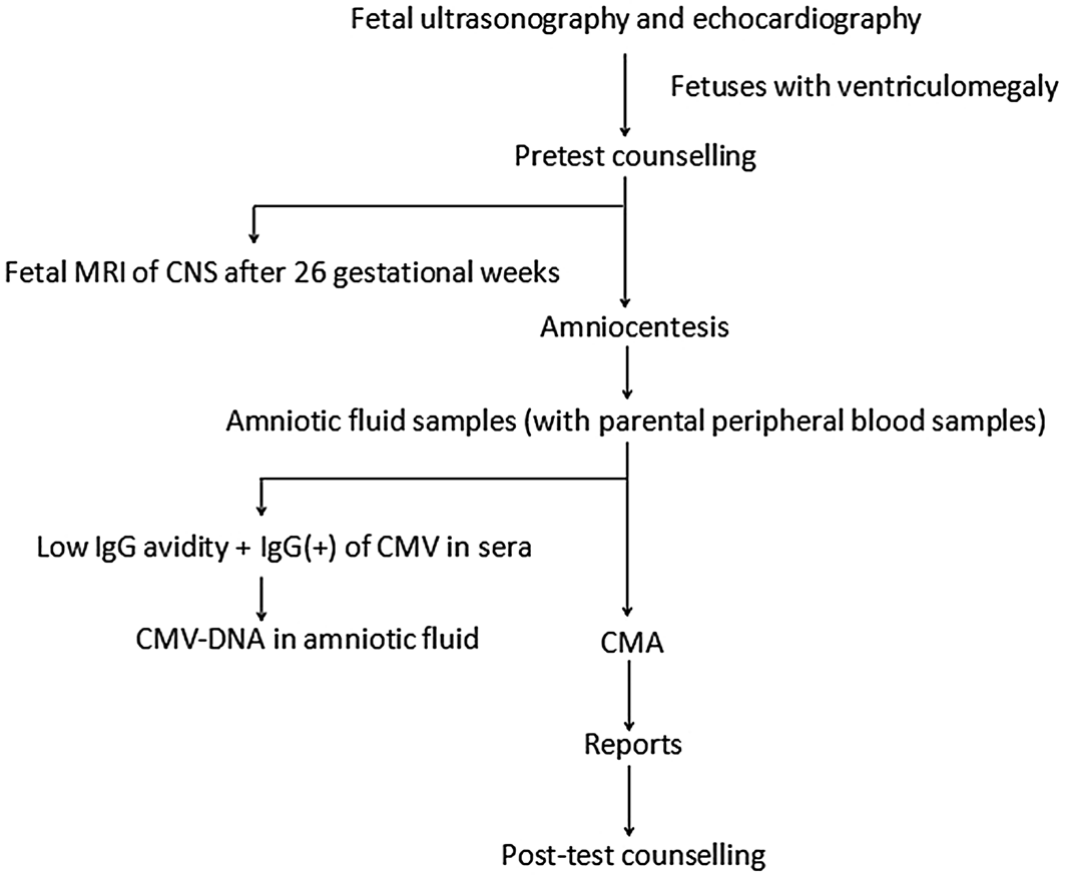
The flow chart of the study.

Between September 2014 and December 2018, a total of 548 singleton pregnancies with foetal ventriculomegaly detected by ultrasound and recruited from the Prenatal Diagnosis Center of West China Second University Hospital, Sichuan University, were included in this study. Pre-test counselling was conducted by trained clinical geneticists. All foetal samples were obtained through amniocentesis for CMA testing at a GA from 22 to 34^+5^ weeks, which was calculated according to crown-rump length in the first trimester. Clear amniotic fluid samples were tested directly, whereas blood-stained amniotic fluid samples were cultured before CMA testing. Additionally, the peripheral blood samples of the parents were obtained to confirm the inherited or de novo CNVs of the foetuses and determine the clinical significance. The flow chat is shown in Fig. 2.

Lateral ventricle atria were measured on an axial plane at the level of the thalamic nuclei, just below the standard image, to measure the biparietal diameter (BPD). When foetal ventriculomegaly were suspected, ultrasonography and echocardiography were performed by two foetal sonographers (Voluson E8, GE Medical Systems, Zipf, Austria) to confirm the diagnosis in the hospital, as per the guidelines of the International Society of Ultrasound Obstetrics and Gynaecology^5, 32^. Furthermore, foetal MRI of the CNS was recommended after 26 weeks GA^5, 33^.

There were 368 (67.15%) foetuses with unilateral ventriculomegaly and 180 (32.85%) with bilateral ventriculomegaly. The foetuses were categorized into 2 groups: isolated ventriculomegaly (n = 294) and non-isolated ventriculomegaly (n = 254). Non-isolated ventriculomegaly anomalies included structural anomalies in other system(s) or sonographic soft markers. The soft markers included intracardiac echogenic focus, increased nuchal fold, hyperechogenic bowel, mild pyelectasis, short femur, short humerus, aberrant right subclavian artery, absent or hypoplastic nasal bone, single umbilical artery, choroid plexus cysts, and enlarged cisterna magna^34, 35^. Concomitantly, the foetuses were also categorized into the following 3 groups according to the atrial diameter of the lateral ventricle: mild ventriculomegaly (10–12 mm) (n = 439), moderate ventriculomegaly (13–15 mm) (n = 83), and severe ventriculomegaly (> 15 mm) (n = 26).

Written informed consent was obtained from all gravidas before testing. The study was approved by the Medical Ethics Committee of West China Second University Hospital, Sichuan University. The research was conducted in accordance with the relevant guidelines and clinical norms.

### Chromosomal microarray analysis

Genomic DNA of foetuses and parents was extracted from amniotic fluid and peripheral blood samples using the QIAamp DNA Blood Mini Kit (Qiagen, Valencia, CA, USA) and subsequently subjected to SNP array analysis using the CytoScan 750 K Array (Thermo Fisher Scientific, Santa Clara, CA, USA). The procedures were conducted according to the manufacturers’ instructions with 250 ng of gDNA, as described in our previous publication^36^. The molecular karyotype analysis was performed using Chromosome Analysis Suite (ChAS) v4.1 (ThermoFisher Scientific). The GRCh37 (hg19) genome was used for annotation. CNVs larger than 100 kb or that affected more than 50 contiguous probes were considered.

The detected CNVs were systematically evaluated for clinical significance based on scientific literature review and the following public databases: PubMed (https://www.ncbi.nlm.nih.gov/pubmed/), UCSC Genome Browser (https://genome.ucsc.edu/), Online Mendelian Inheritance in Man (OMIM) (https://omim.org/), Database of Genomic Variants (https://dgv.tcag.ca/dgv/app/home), DECIPHER (https://decipher.sanger.ac.uk/browser), GeneReviews (https://www.ncbi.nlm.nih.gov/books/NBK1116/), ClinVar (https://www.ncbi.nlm.nih.gov/clinvar/), and ClinGen (https://www.ncbi.nlm.nih.gov/projects/dbvar/clingen/). The clinical significances of detected CNVs were classified into 5 categories: pathogenic CNVs (P CNVs), likely pathogenic CNVs (LP CNVs), VUS, likely benign CNVs, and benign CNVs. This classification was conducted according to the guidelines of the American College of Medical Genetics and Genomics as follows: (1) initial assessment of genomic content, (2) established triplosensitive, haploinsufficient, or benign genes or genomic regions, (3) gene number, (4) cases from published literature, public databases, and/or internal lab data, and (5) inheritance patterns/family history for patient^37^. The CNVs defined as likely benign or benign were not reported.

### Fluorescence in situ hybridization analysis (FISH)

This procedure was also described in our previous study^36^.

### Chromosomal karyotyping

This procedure was also described in our previous study^36^.

### lgM and lgG antibodies of TORCH agents detection

This procedure was described in Supplementary Methods.

### Polymerase chain reaction (PCR) test for CMV-DNA

This procedure was also described in Supplementary Methods.

### Clinical follow-up assessments

Clinical follow-up assessments via telephone were scheduled and conducted from 6 months to 3 years after birth to evaluate pregnancy outcomes and circumstances after birth, including postnatal imaging and developmental details, etc.

### Statistical analysis

Statistical analysis was performed using SPSS software, version 24.0. Continuous data were presented as the means ± standard deviations. Comparison of continuous variables between groups was performed using the two-tailed Student’s t test and analysis of variance using Levene’s test. If the distribution was extremely skewed, a nonparametric two–tailed Mann–Whitney U test was used. Comparison of categorical variables between groups was performed using the Χ^2^ or Fisher exact test. A value of *P* < 0.05 was considered to indicate statistical significance.

## Supplementary information

Supplementary information.

## References

[1] Scala C (2017). Perinatal and long-term outcomes in fetuses diagnosed with isolated unilateral ventriculomegaly: Systematic review and meta-analysis. Ultrasound. Obstet. Gynecol..

[2] Griffiths PD, Reeves MJ, Morris JE, Mason G, Russell SA, Paley MN, Whitby EH (2010). A prospective study of fetuses with isolated ventriculomegaly investigated by antenatal sonography and in utero MR imaging. AJNR. Am. J. Neuroradiol..

[3] Hannon T, Tennant PW, Rankin J, Robson SC (2012). Epidemiology, natural history, progression, and postnatal outcome of severe fetal ventriculomegaly. Obstet. Gynecol..

[4] Kennelly MM, Cooley SM, McParland PJ (2009). Natural history of apparently isolated severe fetal ventriculomegaly: Perinatal survival and neurodevelopmental outcome. Prenat. Diagn..

[5] Society for Maternal-Fetal Medicine (SMFM), Fox, N. S., Monteagudo, A., Kuller, J. A., Craigo, S. & Norton, M. E. Mild fetal ventriculomegaly: Diagnosis, evaluation, and management. *Am. J. Obstet. Gynecol.* **219**, B2–B9 (2018). 10.1016/j.ajog.2018.04.039 29705191

[6] Salomon LJ (2006). Third-trimester fetal MRI in isolated 10- to 12-mm ventriculomegaly: Is it worth it?. BJOG.

[7] Melchiorre K, Bhide A, Gika AD, Pilu G, Papageorghiou AT (2009). Counseling in isolated mild fetal ventriculomegaly. Ultrasound. Obstet. Gynecol..

[8] Chang Q (2020). Prenatal detection of chromosomal abnormalities and copy number variants in fetuses with ventriculomegaly. Eur. J. Paediatr. Neurol..

[9] Donnelly JC, Platt LD, Rebarber A, Zachary J, Grobman WA, Wapner RJ (2014). Association of copy number variants with specific ultrasonographically detected fetal anomalies. Obstet. Gynecol..

[10] Shaffer LG (2012). Detection rates of clinically significant genomic alterations by microarray analysis for specific anomalies detected by ultrasound. Prenat. Diagn..

[11] Sun L (2015). Prenatal diagnosis of central nervous system anomalies by high-resolution chromosomal microarray analysis. Biomed. Res. Int..

[12] Hu P (2017). Copy number variations with isolated fetal ventriculomegaly. Curr. Mol. Med..

[13] Duan HL (2019). The application of chromosomal microarray analysis to the prenatal diagnosis of isolated mild ventriculomegaly. Taiwan. J. Obstet. Gynecol..

[14] Yi JL, Zhang W, Meng DH, Ren LJ, Yu J, Wei YL (2019). Epidemiology of fetal cerebral ventriculomegaly and evaluation of chromosomal microarray analysis versus karyotyping for prenatal diagnosis in a Chinese hospital. J. Int. Med. Res..

[15] Wang Y, Hu P, Xu Z (2018). Copy number variations and fetal ventriculomegaly. Curr. Opin. Obstet. Gynecol..

[16] Gaglioti P, Oberto M, Todros T (2009). The significance of fetal ventriculomegaly: Etiology, short- and long-term outcomes. Prenat. Diagn..

[17] Bromley B, Frigoletto FD, Benacerraf BR (1991). Mild fetal lateral cerebral ventriculomegaly: Clinical course and outcome. Am. J. Obstet. Gynecol..

[18] Sethna F, Tennant PW, Rankin J, Robson C, S.  (2011). Prevalence, natural history, and clinical outcome of mild to moderate ventriculomegaly. Obstet. Gynecol..

[19] Li Z (2017). Application of chromosome microarray analysis for the delineation of pathogenesis for fetal ventriculomegaly. Zhonghua. Yi. Xue. Yi. Chuan. Xue. Za. Zhi..

[20] Zhang Z (2015). Chromosomal microarray analysis for lateral ventriculomegaly in fetus. Zhonghua. Yi. Xue. Yi. Chuan. Xue. Za. Zhi..

[21] Gezer C (2014). Chromosome abnormality incidence in fetuses with cerebral ventriculomegaly. Obstet. Gynaecol..

[22] Etchegaray A, Juarez-Peñalva S, Petracchi F, Igarzabal L (2020). Prenatal genetic considerations in congenital ventriculomegaly and hydrocephalus. Childs. Nerv. Syst..

[23] Riedijk S (2014). The psychological challenges of replacing conventional karyotyping with genomic SNP array analysis in prenatal testing. J. Clin. Med..

[24] Joint Committee on Genomics in Medicine. Recommendations for the use of chromosome microarray in pregnancy (2015).

[25] Devaseelan P, Cardwell C, Bell B, Ongm S (2010). Prognosis of isolated mild to moderate fetal cerebral ventriculomegaly: A systematic review. J. Perinat. Med..

[26] Pagani G, Thilaganathan B, Prefumo F (2014). Neurodevelopmental outcome in isolated mild fetal ventriculomegaly: Systematic review and meta-analysis. Ultrasound. Obstet. Gynecol..

[27] Beeghly M (2010). Neurodevelopmental outcome of fetuses referred for ventriculomegaly. Ultrasound. Obstet. Gynecol..

[28] Pasquini L (2014). The utility of infection screening in isolated mild ventriculomegaly: An observational retrospective study on 141 fetuses. Prenat. Diagn..

[29] Anselem O (2011). Fetal tumors of the choroid plexus: Is differential diagnosis between papilloma and carcinoma possible?. Ultrasound. Obstet. Gynecol..

[30] Finckh U, Schröder J, Ressler B, Veske A, Gal A (2009). Spectrum and detection rate of L1CAM mutations in isolated and familial cases with clinically suspected L1-disease. Am. J. Med. Genet..

[31] Kousi M, Katsanis N (2016). The genetic basis of hydrocephalus. Annu. Rev. Neurosci..

[32] International Society of Ultrasound in Obstetrics & Gynecology Education Committee. Sonographic examination of the fetal central nervous system: guidelines for performing the 'basic examination' and the 'fetal neurosonogram'. *Ultrasound. Obstet. Gynecol*. 29, 109–116 (2007). 10.1002/uog.3909 17200992

[33] Parazzini C (2012). Is fetal magnetic resonance imaging indicated when ultrasound isolated mild ventriculomegaly is present in pregnancies with no risk factors?. Prenat. Diagn..

[34] Agathokleous M, Chaveeva P, Poon LC, Kosinski P, Nicolaides KH (2013). Meta-analysis of second-trimester markers for trisomy 21. Ultrasound. Obstet. Gynecol..

[35] Van den Hof MC, Wilson RD (2005). 2005 Diagnostic Imaging Committee, Society of Obstetricians and Gynaecologists of Canada; Genetics Committee, Society of Obstetricians and Gynaecologists of Canada. Fetal soft markers in obstetric ultrasound. Obstet. Gynaecol. Can..

[36] Hu T (2019). Prenatal diagnosis of chromosomal aberrations by chromosomal microarray analysis in fetuses with ultrasound anomalies in the urinary system. Prenat. Diagn..

[37] Riggs ER (2020). Technical standards for the interpretation and reporting of constitutional copy-number variants: A joint consensus recommendation of the American College of Medical Genetics and Genomics (ACMG) and the Clinical Genome Resource (ClinGen). Genet. Med..

