# Supplementary material for: Prenatal diagnosis of chromosomal aberrations by chromosomal microarray analysis in foetuses with ventriculomegaly
Source: Sci Rep. 2020 Nov 27;10:20765. doi: 10.1038/s41598-020-77400-8 (PMC7699619; doi:10.1038/s41598-020-77400-8)
Supplement: Supplementary file 1 — Supplementary information. [file 41598_2020_77400_MOESM1_ESM.pdf]

Prenatal Diagnosis of Chromosomal Aberrations by Chromosomal Microarray Analysis in Fetuses with Ventriculomegaly

Jiamin Wang[1,2], Zhu Zhang[1,2], Qinqin Li[1,2], Hongmei Zhu[1,2], Yi Lai[1,2], Wei Luo[1,2], Shanling Liu[1,2], He Wang[1,2], Ting Hu[1,2]*

SUPPLEMENTARY METHODS

IgM and IgG antibodies of TORCH agents detection

All gravidas who underwent amniocentesis performed TORCH screening for serum IgM and IgG antibodies of TOX, Rubella virus (RV), CMV, and Herpes Simplex Virus (HSV) by Chemiluminescent Micropaticle Immuno Assay (CMIA) kits (DiaSorin, Saluggia, VC, Italy) using LIAISON analyzer (DiaSorin) according to the manufacturer's instructions. The sera tested for CMIA were based on the capture principle for IgM antibody and the indirect principle for IgG antibody, respectively [1].

In gravidas with positive CMV or TOX IgM results, serum IgG avidity testing was further applied by LIAISON CMV IgG Avidity II Kit or LIAISON XL Toxo IgG Avidity Kit (DiaSorin, Saluggia, VC, Italy) using LIAISON analyzer (DiaSorin) according to the manufacturer's instructions.

Polymerase chain reaction (PCR) test of CMV-DNA

Until now, as the only PCR test of CMV was approved for clinical practice by the China Food and Drug Administration (CFDA), for gravidas with low IgG avidity and positive IgM of CMV, quantification of CMV-DNA was performed on clear amniotic fluid samples by Real-time fluorescence quantitative PCR reaction Kit (DaAn Gene Co., Ltd., ZhongShan, China). The boiling method (100 °C for 10 min) was used to extract DNA from amniotic fluid samples. PCR program on ABI Prism 7500 Sequence Detection System (Thermo Fisher) was performed as follows: 93°C for 2

min, followed by 10 cycles of 93°C for 45 s, 55°C for 60 s, then 30 cycles of 93°C for 30 s, 55°C for 45 s.

REFERENCES

[1] Wang, L. C., Yan, F., Ruan, J. X., Xiao, Y., Yu, Y. TORCH screening used appropriately in China?—three years results from a teaching hospital in northwest China. *BMC. Pregnancy. Childbirth.* 19, 484 (2019).

SUPPLEMENTARY TABLES

Supplementary Table S1 Demographic and clinical characteristics of the gravidas

Characteristics	n	*P*	
**Age (mean ± SDs)**	28.68 ± 4.49		
**Lateral of VM**			
Unilateral (n=368)	28.42 ± 4.36	0.056	
Bilateral (n=180)	29.21 ± 4.72		
**Ultrasound findings**			
Isolated VM (n=294)	28.54 ± 4.43	0.456	
Non-isolated VM (n=254)	28.83± 4.55		
**Degrees of VM**			
Mild (n=439)	28.75 ± 4.41	0.397	(Mild vs. Moderate)
Moderate (n=83)	28.30 ± 4.76	0.911	(Mild vs. Severe)
Severe (n=26)	28.65± 5.10	0.746	(Moderate vs. Severe)
**Parity (0/ 1/ 2)**	334/ 187/ 27		
**Lateral of VM**			
Unilateral (n=368)	228/ 124/ 16	0.604	
Bilateral (n=180)	106/ 63/ 11		
**Ultrasound findings**			
Isolated VM (n=294)	149/ 95/ 10	0.242	
Non-isolated VM (n=254)	185/ 92/ 17		
**Degrees of VM**			
Mild (n=439)	276/ 144/ 19		
Moderate (n=83)	49/ 29/ 5	0.054	
Severe (n=26)	9/ 14/ 3		
**Abnormal Pregnancy History**	25		
**Lateral of VM**			
Unilateral (n=368)	16	0.731	
Bilateral (n=180)	9		
**Ultrasound findings**			
Isolated VM (n=294)	12	0.562	
Non-isolated VM (n=254)	13		
**Degrees of VM**			
Mild (n=439)	18		
Moderate (n=83)	5	0.547	
Severe (n=26)	2		

SD: standard deviations; VM: ventriculomegaly
